# Alteration of fear behaviors in sleep-deprived adolescent rats: increased fear expression and delayed fear extinction

**DOI:** 10.1080/19768354.2021.1902854

**Published:** 2021-03-22

**Authors:** Taesub Jung, Jihyun Noh

**Affiliations:** Department of Science Education, Dankook University, Yongin-si, Republic of Korea

**Keywords:** Adolescence, fear conditioning, fear extinction, hyperactivity, sleep

## Abstract

Disruption of sleep due to acute or chronic stress can lead to changes in emotional memory processing. Sleep disturbances are highly prevalent in post-traumatic stress disorder (PTSD), but still, the contribution of sleep deprivation on the susceptibility to PTSD has received little attention. To determine whether rapid eye movement sleep deprivation (SD) alters the development of fear expression or fear-associated memory impairment in adolescent rats, we performed animal emotional behavior tests using an SD animal model with the flowerpot technique. SD rats showed an increase in locomotor activity frequency and a decrease in sucrose consumption compared to control rats. An increase in freezing behavior during shock trials was observed in SD rats. Noticeably, it was observed that when applying the SD condition after fear stimuli exposure, fear extinction was delayed more in SD rats than in control rats. Overall, these results indicate that SD in adolescent rats leads to increased locomotor activity and anhedonic behavior, as well as increased fear expression and delayed fear extinction, suggesting that SD would lead to increased severity of PTSD-like phenotype.

## Introduction

Disruption of sleep by acute or chronic stress may lead to alterations in emotional memory processing and, thereby, contribute to psychiatric illnesses such as post-traumatic stress disorder (PTSD) (Battle 2013). Epidemiological and prospective studies show that sleep disturbances that exist prior to traumatic exposure or that occur immediately after traumatic exposure are strong risk factors for poor psychiatric outcomes, including PTSD, anxiety disorders, and mood disorders (Breslau et al. 1996; Bryant et al. 2010).

Rapid eye movement (REM) sleep is crucial for maintaining a regular mood in humans. The transition to REM sleep is accompanied by a rapid decrease in monoaminergic tone (serotonin, norepinephrine, and dopamine) and a concomitant increase in cholinergic tone (Pace-Schott and Hobson 2002). This suggests the possibility of REM SD affecting emotions. Several previous studies have reported the effects of REM SD on alterations in emotional behavior; however, the results are still controversial.

Anxiogenic behaviors were observed not only in 72-h REM sleep-deprived male EPM-M1 mice and in 48-h REM sleep-deprived female BALB/c mice, but also in chronic REM sleep-deprived male Wistar rats during 21 consecutive days for 18 h/day (Gonzalez-Castañeda et al. 2016; Da Silva Rocha-Lopes et al. 2018). However, anxiolytic behaviors were observed in 24-h REM sleep-deprived Wistar rats and naive male albino mice (Pokk et al. 1996; Pokk and Vali 2001; Silva et al. 2004; Parsa et al. 2016). Depressive behavior was observed in 120-h REM sleep-deprived C57BL/6 mice; however, anti-depressive behavior also occurred in mice with 72-h REM SD (De Oliveira et al. 2004; Zhen et al. 2017). In the sucrose consumption test to measure taste aversion, a reduction in sucrose intake in the 48-h REM sleep-deprived male Wistar rats was reported when compared with the control rats (Pezzato et al. 2009). However, in the forced swimming test to measure depressive behavior, an increase in swimming activity was examined following 24-h REM SD (Asakura et al. 1993).

Sleep-deprived C57BL/6 mice by gentle stroking selectively impairs memory consolidation for contextual fear conditioning (Graves et al. 2003), but REM SD by flowerpot technique shows normal retention and extinction of a contextual conditioning task in Sprague–Dawley rat (Silvestri 2005). REM SD reduces cued fear extinction and induces impairment of conditioned fear responses, as seen in many experiments (Graves et al. 2003; Ruskin et al. 2004; Yang et al. 2012; Hunter 2014; Qureshi and Jha 2017; Straus et al. 2017), but on the contrary, it is suggested that prolonged REM SD in adult male Sprague–Dawley rats impairs contextual fear learning but not cued fear learning (Ruskin et al. 2004). The phenomenon of insomnia in PTSD is likely to result in the inability of these negative memories to be easily eliminated (Cohen et al. 2012). As the fear effect by REM SD could be different depending on the timing of fear conditioning and the type of fear conditioning tests, it would be necessary to investigate the various REM SD-induced effects in fear expression and fear extinction based on exposure before and after fear conditioning and effects in contextual and cued fear expression tests.

Notably, most patients with schizophrenia, bipolar depression, male obsessive-compulsive disorder, and panic attacks develop the psychiatric disorder during puberty, with unipolar depression having a high occurrence among adolescents (Garcia-Rill 1997). Moreover, like insomnia, high rates of sleep problems occur among children with psychiatric disorders (Ivanenko et al. 2004; Gregory and Sadeh 2016). Many adult psychiatric patients indicate that their sleep problems originated during childhood (Philip and Guilleminault 2017), and impaired REM sleep regulation during adolescence may lead to major depression, insomnia, Alzheimer’s disease, and Huntington’s disease (Brand and Kirov 2011). However, studies on behavior change related to emotion by REM SD during adolescence are still insufficient.

This study aimed to clarify the contribution of SD on the susceptibility to PTSD-like phenotype. To determine this issue, a 48-h REM SD rat model via the flowerpot technique was proposed, particularly in an adolescence period that is more developmentally critical and more sensitive to SD (Shaffery et al. 2006; Yang et al. 2012).

## Materials and methods

### Animals

**Figure 1.**
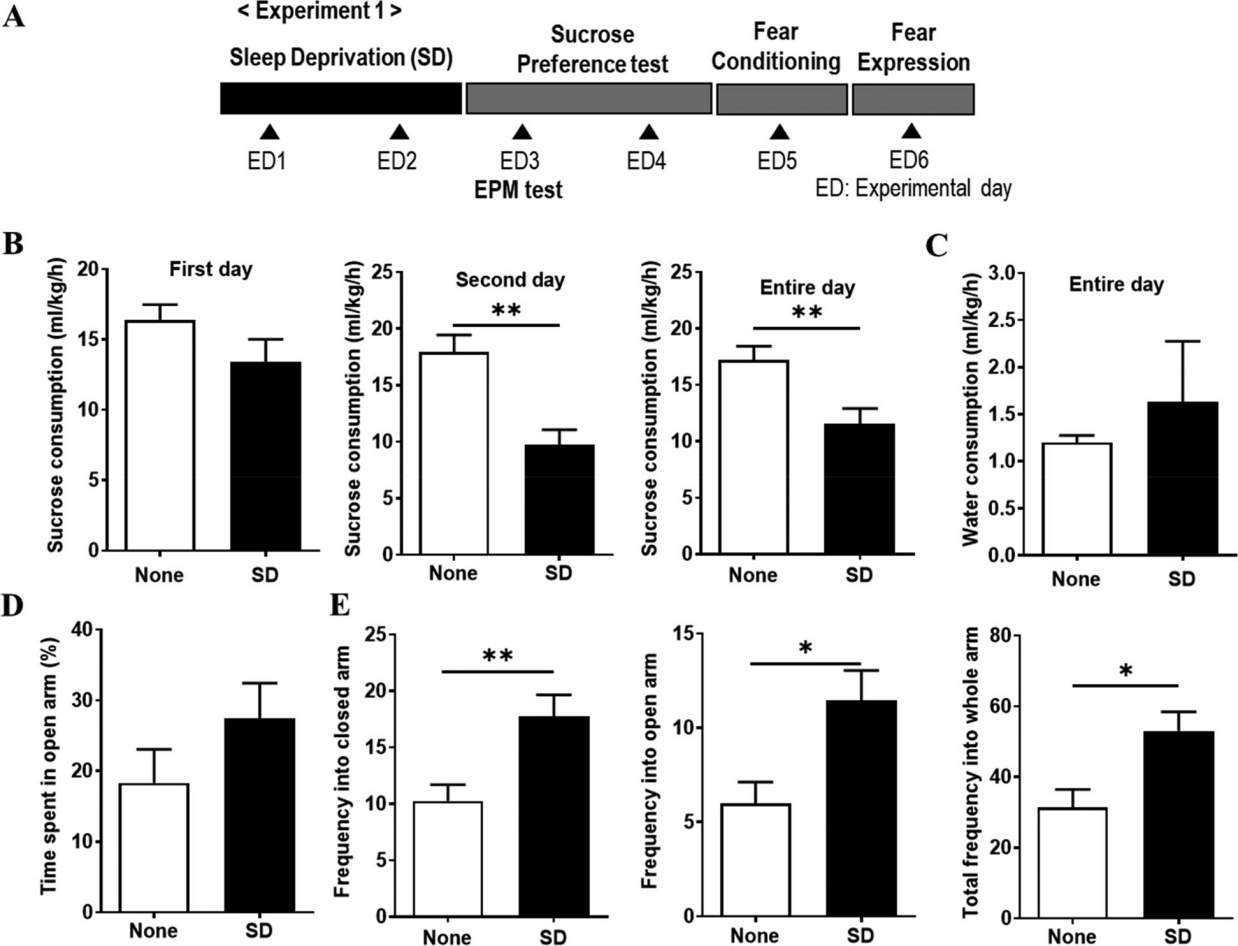
Decrease in sucrose consumption and increase in movement frequency in the arms by sleep deprivation (SD). (A) Schematic timeline of Experiment 1 procedures. Rats were deprived of sleep (sleep deprivation [SD]) or not deprived of sleep (None) for 48 h. After SD, an elevated plus-maze (EPM) test, sucrose consumption test, and a fear memory (fear conditioning and fear expression) test was carried out. ED, experimental day. (B) All consumption was calculated as their liquid consumption divided by their weight per hour. Sucrose consumption (**P < 0.01, None *vs.* SD; unpaired *t*-test). (C) Water consumption. (D) Time spent in the open arm during 10 min. Movement frequency into the closed arm, the open arm, and the whole arm during 10 min (*P < 0.05, **P < 0.01, None *vs.* SD; unpaired *t*-test).

Adolescent female Sprague–Dawley rats (3 weeks, weight 40–80 g) were obtained from Orient Bio Inc. (Seongnam, South Korea). All experiments were conducted according to the Dankook University Ethics Committee’s Guidelines for the Care and Use of Laboratory Animals (DKU-17-033). The rats were housed in Plexiglas cages (46 × 23 × 20 cm) with wooden bedding with a 12:12 h light–dark cycle (lights on at 9 AM). Except for the SD test, other tests and the measurement of body weight and water consumption proceeded till almost 1 PM Food and water were provided ad libitum. The experiments were conducted in a sound-insulated room with controlled temperature (23 ± 1°C) and humidity (45 ± 5%). The experimental schematic timeline is shown in Figure 1(A) (Experiment 1), Figure 3(A) (Experiment 2) and Figure 4(A) (Experiment 3). In experiment 1, the elevated plus-maze (EPM), the sucrose consumption test, and the fear memory test after a 48-h SD test were sequentially examined. In experiment 2, a 48-h SD test after fear conditioning was performed, and then the contextual fear expression tests were examined. In experiment 3, a 48-h SD test after fear conditioning was performed, and then the cued fear expression and extinction test were examined. The rats were assigned to one of the following two treatment groups: (1) the None group, which was exposed to a controlled condition without water (*n* = 21; experiment 1, *n* = 8; experiment 2, *n* = 7; experiment 3, *n* = 6), (2) the SD group, which was deprived of sleep using the flowerpot technique with water (*n* = 20; experiment 1, *n* = 7; experiment 2, *n* = 7; experiment 3, *n* = 6).

### REM sleep deprivation (SD) model

The REM SD model was induced using the inverted flowerpot technique (Mendelson et al. 1974). Individual rats were placed on top of platforms (6.5 cm in diameter, 4 cm in height) surrounded by water located in Plexiglas cages (46 × 23 × 20 cm). Food and water were provided through a grid on the top of the cage. Sleep was disturbed when the body contacted the water as a result of muscle atonia associated with REM sleep onset, thus awakening the animal. For the sleep-deprived rats, the base of the cage was submerged under 1 cm of water for two consecutive days (48 h) (Youngblood et al. 1997; May et al. 2005; Wei et al. 2007; Li et al. 2009; Aleisa et al. 2011; Colavito et al. 2013; Hajali et al. 2015). Some reports suggest the result of reduced REM-related EEG by flowerpot technique (Endo et al. 1997; Mueller et al. 2008).

### Sucrose consumption test

For acclimation to the sucrose solution, 1% sucrose (Sigma, St. Louis, MO, USA) solution and tap water contained in two bottles were provided to the individual rats for 24 h. The volume of both the sucrose solution and tap water was the same (250 mL), and the remainder of both the sucrose solution and tap water were checked to measure consumption. The initial positions of the two bottles were randomized to prevent a positional preference, and after 24 h, the positions of the bottles were switched to avoid adjustment to the position of the liquids.

### Elevated plus-maze (EPM) test

The EPM test consisted of two open arms (50 × 10 cm) and two closed arms (50 × 10 × 40 cm) with a central platform (10 × 10 cm), which was elevated 50 cm above the floor. The open arms had a very short (0.5 cm) wall to decrease the number of falls, and the closed arms had a high (40 cm) wall. The time spent in the open or closed arms was recorded for 10 min using a camcorder (HMX-H304BD, Samsung, South Korea). The camcorder was placed 150 cm above the floor with a view of the plus-maze. The frequency of open or closed arm entries (number of open or closed arm entries) and the time spent in the open or closed arms (time spent in open or closed arms / time spent in [open + closed arms] × 100) was calculated for each rat.

### Fear conditioning test

After a 24-h acclimation period, all rats were introduced into a shock box (Jeung Do Bio & Plant, Seoul, South Korea) consisting of two equal-sized rooms (24.5 × 24.5 × 23.5 cm) separated by a transparent window. Before commencing conditioning exposure, there was an initial 2-min baseline period to adapt to a black room with an opaque plate that prevented passage to another room. During the 8-min conditioning period, all rats were exposed to eight 3-s light cues that co-terminated with a 1-mA electric foot shock for 1 s, delivered through the floor, which consisted of a 22-square grid (0.4 cm in diameter with intervals of 1.1 cm). Four cues were randomly arranged at 30 s, and the other four were randomly arranged at 90 s to learn that the electrical stimulus given after the cue was based on the interval, not the pattern. The light cue was produced by a 150-lx lamp placed on the ceiling of the shock box black room, in an environment where external lights were extinguished. Freezing behaviors were recorded using a camcorder (HMX-H304BD) and analyzed as the fear response of the rats. Freezing behavior was defined as the absence of all observable movements of the skeleton and the vibrissae, except for those related to respiration (Hashimoto et al. 1999). Freezing behavior was blindly scored by two independent observers (one of whom did not know the experimental grouping of the animals). We compared the ratio of freezing behavior across different lengths of time by dividing the eight stimuli given by random time differences into eight blocks and calculated the ratio of freezing behavior in each block.

### Contextual and cued fear expression test and fear extinction test

For experiment 1, the fear expression tests were conducted at 24-h intervals from the fear conditioning test and performed for 10 min as the same pattern as that of the fear conditioning test. The rats were individually re-introduced into the same shock box and exposed only to the light cue without electric foot shocks. After 2-min exploration without a cue in the black room, in the 8-min stimulus period, the rats were exposed to a cue as a randomized different pattern consisting of four 30-s intervals and four 90-s intervals. Different cue patterns allow the target to focus more on the cue themselves, rather than on the intervals.

The contextual fear expression test was performed after 48-h REM SD test and the rats were individually re-introduced into the shock box. The freezing behavior duration was estimated during a 2-min exploration in the shock box to observe response of fear memory when exposed to the same contextual situation before (Experiment 2).

The cued fear expression test was performed after 48-h REM SD test and the rats were individually re-introduced into the shock box with different contextual condition. When the conditioned stimulus (CS) is subsequently presented repeatedly without the unconditioned stimulus (US), extinction of the cue-conditioned response typically takes place. However, behavior studies of extinction suggest that it is rather than erasing the CS-US association or processing ‘unlearning’, extinction represents a process of the new learning of fear inhibition or a formation of extinction memory, signifying CS-no US (Phelps et al. 2004; Milad and Quirk 2012). The cued fear extinction tests were conducted at 24-h intervals twice over two days and rats were performed for 10 min as the same pattern as that of the fear conditioning test without foot shock (Experiment 3). The freezing behavior duration was estimated during an 8-min stimulus period in the shock box to compare each period’s freezing and measure delay of fear memory extinction gradually.

### Data analysis

The data were analyzed using GraphPad Prism 8 software (GraphPad Software, Inc., San Diego, CA, USA), and statistical descriptions were made using the mean ± standard error mean. Statistical significance for the two groups was evaluated using an unpaired *t*-test for EPM, and sucrose test. Statistical significance for multiple groups was evaluated using a two-way ANOVA followed by Bonferroni’s multiple comparisons test for the duration of freezing behavior during the fear conditioning, expression, and extinction test. For all statistical tests, the rejection criterion was set to *P* < 0.05.

## Results

### Decreased sucrose consumption and increased movement frequency after SD

In all experiments (experiment 1, 2, and 3), the reduction of water consumption in the SD group was observed during the 48-h SD period compared with the none group, and there were no significant differences in water consumption during the remainder of the test and in body weight between the SD group and the none group (Table 1).

**Table 1.** Changes in water consumption and in body weight.

*Experiment 1*								
ED		0	1 (SD)	2 (SD)	3	4	5	6
Body weight (g)	None	61.25 ±16.18	69.76 ±16.45	76.25 ±16.98	82.15 ±17.62	89.73 ±17.63	96.68 ±17.62	104.10±18.09
	SD	58.56 ±14.39	61.50 ±15.41	66.51±16.27	74.47 ±17.73	82.69 ±18.08	90.06 ±19.06	97.04 ±19.41
Water Consumption (ml)	None		11.34±2.87	8.92 ±1.42	1.15 ±0.37	1.24 ±0.32	9.81 ±1.95	8.78 ±2.22
	SD		5.62±1.44***	2.22±0.57***	1.24 ±0.97	2.02 ±2.27	8.34 ±0.48	7.59 ±0.61
*Experiment 2*								
ED		1	2 (SD)	3 (SD)	4			
Body weight (g)	None	58.51±8.10	63.26 ±8.32	69.61 ±8.22	76.89 ±8.83			
	SD	61.91 ±7.02	63.43 ±8.07	69.40 ±7.29	76.84 ±7.52			
Water Consumption (ml)	None		9.59 ±1.04	9.80 ±1.11	10.00 ±0.72			
	SD		3.27±2.31***	3.79±1.92***	7.05 ±2.85			
*Experiment 3*								
ED		1	2 (SD)	3 (SD)	4			
Body weight (g)	None	52.37 ±2.24	57.80 ±1.97	63.05 ±3.01	69.02 ±3.72			
	SD	47.87 ±3.49	51.82 ±3.09	54.52 ±2.71	60.98 ±2.44			
Water Consumption (ml)	None		15.51 ±1.19	11.90 ±1.27	13.58 ±1.39			
	SD		9.96 ±2.18***	4.25±0.94***	14.83 ±1.28			

Two-way ANOVA, Bonferroni post-hoc tests; ***P < 0.001, None *vs*. SD.

In sucrose consumption test to determine the effects of SD on depressive behavior, sucrose consumption in the SD group was significantly lower than that in the none group on the second day (*P* = 0.0014) and during the entire day of the test (*P* = 0.01) (Figure 1B). Water consumption for the entire day did not significantly differ among groups (Figure 1C).

The EPM test was performed to determine the effects of SD on anxious behavior. No significant differences among the groups were observed in terms of the percentage of time spent in the open arm. However, compared with the none group, the SD group showed a significant increase in the movement frequency in the closed arm (*P* = 0.008), and the open arm (*P* = 0.01), and the total frequency in the whole arm (*P* = 0.01) (Figure 1D). A reduction in the sucrose consumption and an increase in the movement frequency were observed in the SD group, suggesting that SD could induce an increased locomotor behavior and transient depressive behavior.

### Increased fear expression in shock-exposed rats after SD (Experiment 1)

**Figure 2.**
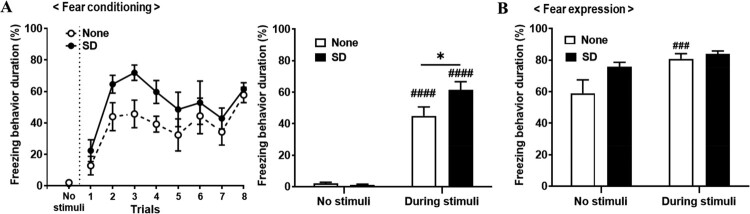
Increased freezing behavior by sleep deprivation (SD) during fear conditioning. (A) *Left*, The comparison of the freezing behaviors in each session over the fear conditioning period between none and SD group. *Right*, Fear acquisition by electric stimuli in the fear conditioning period (two-way ANOVA; ^####^*P* < 0.0001, no stimuli *vs*. during stimuli; *P < 0.05, None *vs.* SD; Bonferroni post-hoc test). (B) *Left*, Freezing behaviors in each session over the fear expression period. *Right*, Fear acquisition by electric stimuli in the fear expression period (two-way ANOVA; ^###^*P* < 0.001, no stimuli *vs*. during stimuli; Bonferroni post-hoc test).

To determine the effects of SD on the fear expressed response after fear conditioning, the freezing behavior in each trial session of each group was measured and compared. The effect of trials (*F* (7, 84) = 8.97, *P* < 0.0001) was only statistically significant, based on a two-way ANOVA (groups, *F* (1, 12) = 3.38, *P* = 0.09; group × trial interaction, *F* (7, 84) = 0.72, *P* = 0.65; Figure 2A, *left*). To compare each no stimuli (before stimulus) and during stimuli (after stimulus), the duration of freezing behavior (%) between the none group and the SD group, the effect of trials (*F* (1, 12) = 207.2, *P* < 0.0001) and the group × trial interaction (*F* (1, 12) = 5.95, *P* = 0.03) were statistically significant, based on a two-way ANOVA (groups, *F* (1, 12) = 3.69, *P* = 0.08, Figure 2A, *right*). Comparing the change in the duration of freezing behavior (%) from no stimuli to during stimuli, a significant increase was seen in both the none group and the SD group (no stimuli *vs*. during stimuli; None, *P* < 0.0001; SD, *P* < 0.0001; Figure 2A, *right*). Under stimulus-exposed conditions, the duration of freezing behavior of the SD group was significantly higher than that of the none group (None *vs*. SD, *P* = 0.01; Figure 2A, *right*).

In the fear expression test, to compare each no stimuli and during stimuli the duration of freezing behavior (%) between the none group and the SD group, the effect of trials (*F* (1, 13) = 19.65, *P* = 0.0007) was only significant, based on a two-way ANOVA (groups, *F* (1, 13) = 2.35, *P* = 0.15; group × trial interaction, *F* (1, 13) = 4.13, *P* = 0.06; Figure 2B). Comparing the change in the duration of freezing behavior (%) from no stimuli to during stimuli, the none group showed a significant increase (no stimuli *vs*. during stimuli, *P* < 0.001; Figure 2B). Under both no stimulus and during stimulus-exposed conditions, there were no significant difference in the duration of freezing behavior between none and SD group (Figure 2B).

### Effects of SD in contextual fear memory after fear conditioning (Experiment 2)

**Figure 3.**
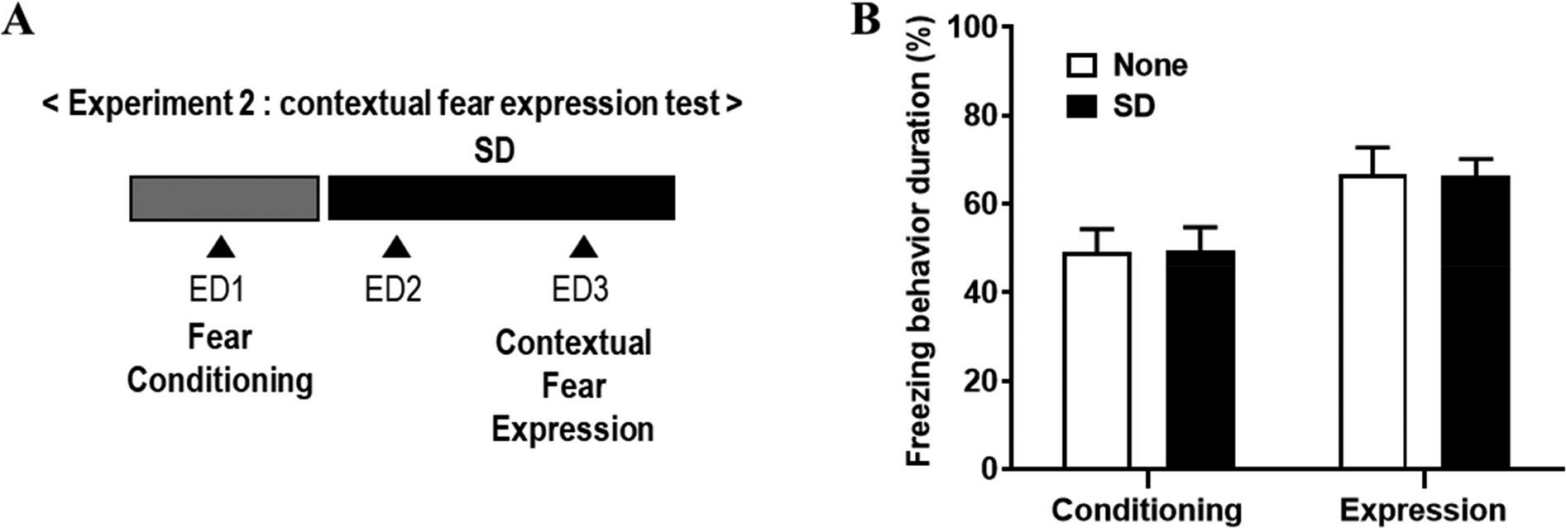
No change of contextual fear expression in shock-exposed rats by SD. (A) Schematic timeline of experiment 2 procedures. Rats were deprived of sleep (SD) or not deprived of sleep (None) for 48 h after fear conditioning, and then, contextual fear expression were carried out. ED, experimental day. (B) Freezing behaviors duration in each session between none and SD group (two-way ANOVA).

In experiment 1, SD exposure induced an increase in freezing behavior during fear conditioning, but in fear memory through fear expression test, no significant difference was found between SD and none group. To clarify the direct effect of SD on fear memory, we examined the contextual and cue fear expression behaviors after fear conditioning (Figure 3).

In experiment 2, the freezing behavior was compared in each session of each group to determine the effects of SD on the contextual fear response after fear conditioning (Figure 3A). The effect of trials (F (1, 12) = 25.1, *P* = 0.003) was only statistically significant (group × trial interaction, F (1, 12) = 0.89, *P* = 0.57; groups, *F* (1, 12) = 0.0001, *P* = 0.99), based on a two-way ANOVA (Figure 3B). Comparing the change in the duration of freezing behavior (%), no significant difference was shown in both the none group and the SD group in contextual fear memory expression (Figure 3B). It seems that effects of SD after fear conditioning was not valid in contextual fear memory.

### Delay of cued fear extinction in shock-exposed rats by SD (experiment 3)

**Figure 4.**
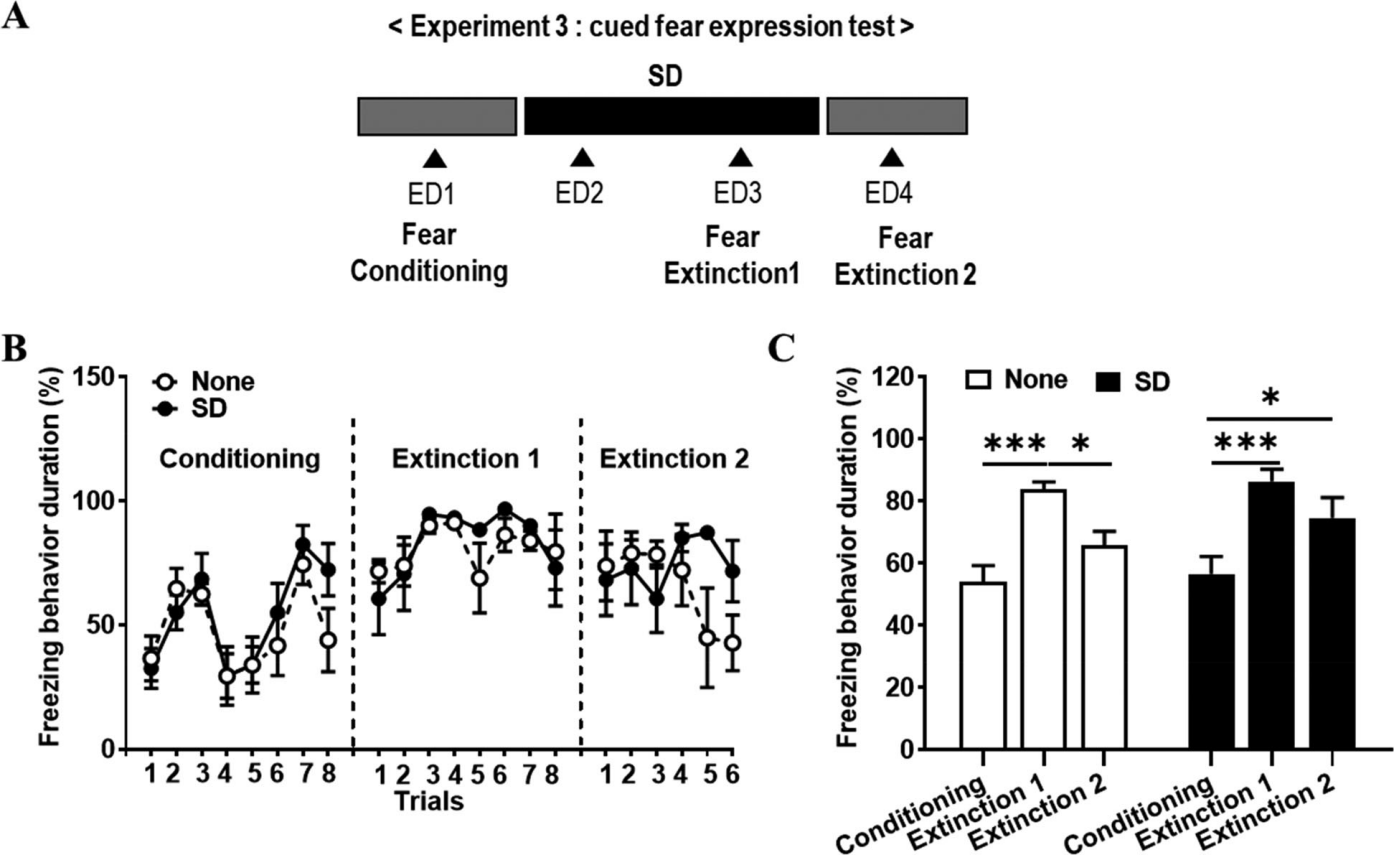
Delay of cued fear extinction in shock-exposed rats by SD. (A) Schematic timeline of experiment 3 procedures. Rats were deprived of sleep (SD) or not deprived of sleep (None) for 48 h after fear conditioning, and then, cued fear extinction 1 and extinction 2 were carried out. (B) The comparison of freezing behaviors in each session, conditioning, extinction 1 and extinction 2 (two-way ANOVA). (C) Freezing behaviors duration in each session between none and SD group (two-way ANOVA; Bonferroni post-hoc test, *P < 0.05, **P < 0.01).

In experiment 3, the freezing behavior was compared in each session of each group to determine the effects of SD on the cued fear response after fear conditioning (Figure 4A). Comparing the change in the duration of freezing behavior (%) between none group and SD group in each conditioning, extinction 1, and extinction 2 session. In conditioning, the effect of trials (*F* (7, 70) = 9.61, *P* < 0.0001) was significant, based on a two-way ANOVA (groups, *F* (1, 10) = 0.37, *P* = 0.56; group × trial interaction, *F* (7, 70) = 1.10, *P* = 0.38; Figure 4B). In extinction 1, the effect of trials (*F* (7, 70) = 2.70, *P* = 0.02) was significant (groups, *F* (1, 10) = 0.28, *P* = 0.61; group × trial interaction, F (7, 70) = 0.63, *P* = 0.73; Figure 4B). In extinction 2, all the effect of trial, groups and group × trial interaction were not significant, based on a two-way ANOVA (trials, *F* (5, 50) = 0.79, *P* = 0.56; groups, *F* (1, 10) = 1.45, *P* = 0.26; group × trial interaction, *F* (5, 50) = 1.85, *P* = 0.12; Figure 4B). Freezing behavior duration in none group significantly increased in the extinction 1 session compared to the conditioning session (*P* = 0.0002) and decreased in the extinction 2 session compared to the extinction 1 session (*P* = 0.0289) (Figure 4C). However, in SD group, freezing behavior duration increased in the extinction 1 compared to the conditioning session (*P* = 0.0002), but there was no significant difference in freezing behavior duration between the extinction 1 and the extinction 2 session (Figure 4C). In addition, in SD group, freezing behavior duration increased in the extinction 2 compared to conditioning session (*P* = 0.0293). It seems that SD after fear conditioning delayed cued fear extinction.

## Discussion

The present study shows that REM SD by flowerpot technique can lead to increased fear response during fear conditioning and delayed fear memory extinction, as well as anhedonic behavior and hyperactive behavior in adolescent female rats.

The most commonly prescribed antidepressant medications increase the monoaminergic tone and reduce REM sleep (Murphy and Peterson 2015). In this study, the sleep-deprived rats showed lower sucrose consumption than the control rats, suggesting that REM SD induces anhedonic depressive behavior (Figure 1B), which is consistent with previous reports that sucrose consumption was lower in female mice after 48-h and 96-h of REM SD compared with control mice (Gonzalez-Castañeda et al. 2016). Thus, depression-related behavior resulting from REM SD can be caused by a problem with the dopaminergic reward system, which can be seen by a decrease in the anhedonic experimental condition, the sucrose test results.

In the EPM test, an anxious animal is expected to spend the most time in the enclosed arms, demonstrating low exploratory and locomotor behaviors (Carobrez and Bertoglio 2005). However, the opposite behavior is expected from an animal with increased impulsivity or mania; it would spend more time in the open arms, demonstrating increased locomotor and exploratory behaviors (Pires et al. 2016). REM SD in rats can cause an increase in locomotor activity (Van Hulzen and Coenen 1981; Pokk and Alexander 1998), which has even been suggested as a model of mania (Gessa et al. 1995). Our finding showed an increased number of entries and time spent in the open arms after SD (Figure 1D). This might be a result of hyperactivity rather than it being due to the anxiolytic effects of sleep perturbations. The reason for the increase in locomotor activity after SD is assumed to be the survival advantage of an increase in boldness when faced with an aversive cue (McBlane and Handley 1994). As a result, the phenomenon of anxiety resulting from REM SD is shown as hyperactivity, which can be seen as a result of enhanced response to survival by the adverse environment given in REM SD.

It was observed that after undergoing SD, fear response increased during exposed to fear conditioning conditions such as PTSD (Figure 2A), suggesting that REM sleep-deprived rats are more sensitive to aversive stimuli (Albert et al., 1970 ). This supports previous findings of enhanced fear acquisition following SD, an SD-associated generalized failure to habituate during fear acquisition, and higher sensitivity to a negative stimulus in sleep-deprived rats (Anderson and Platten 2011; Peters et al. 2014; Feng et al. 2018). This increase in exposure to fear is linked to an increase in vulnerability to SD, which has also been suggested in previous human studies (Lautenbacher et al. 2006). In the case of a short sleep interval, the increased risk of relapse of the disease resulted in increased SD associated with depression (Nutt et al. 2008). Because animal models have confirmed that an increase in vulnerability to this pain disappears with monoamine (Ukponmwan et al. 1986), a study of monoamine will need to verify the mechanism of SD. The results can be linked to earlier results showing differences in sucrose intake in relation to monoamines associated with depression, and we argue that this commonality can cause SD problems in monoaminergic systems.

When experiencing SD after exposure to fear conditioning, there was no effect on contextual fear memory (Figure 3B), which is consistent with previous reports (Silvestri 2005). Meanwhile, we found that cued fear extinction was delayed after SD (Figure 4C), which is consistent with previous study (Kumar and Jha 2012). Previous research has shown that N-methyl-D-aspartate (NMDA) receptors play an important role in fear extinction (Davis 2011), the results of a decrease in the surface stress of the NMDA receptor in hippocampal neurons when sleep is deprived (Chen et al. 2006). It also highlighted the role of NMDA receptors in memory impairment caused by REM SD by checking the results of the decrease in the NMDA–α-amino-3-hydroxy-5-methyl-4-isoxazolepropionic acid (AMPA) ratio and the amplitude of NMDA current after 72-h SD (Li et al. 2009). This suggests that REM deprivation-induced impairment of fear memory extinction is rescued by the NMDA receptor agonist, D-cycloserine (Silvestri and Root 2008). Together with the present findings, it suggests that a REM SD-induced fear extinction delay was induced by NMDA receptor dysfunction, and their underlying mechanism should be studied in the future.

Many REM SD experiments were conducted in male and female rats, but no significant sex differences were found (Koehl et al. 2006; Fernandes-santos et al. 2012; Baratta et al. 2018; Carter et al. 2019; Oyola et al. 2019). However, in some reports, it suggest that female rats are more vulnerable to REM sleep deprivation-induced cognitive impairments than male rats (Hajali et al. 2012) and females show a greater increase in anxiety as a result of sleep deprivation than males (Gonzalez-Castañeda et al. 2016). In addition, acquisition of conditioned fear in woman with PTSD is reported to be enhanced than male (Shansky 2015).

In early adolescent rats, REM SD has been shown to lead to unstable long-term potentiation and low glutamatergic signals, suggesting that REM sleep plays a crucial role in the development of the hippocampus (Lopez et al. 2008). In addition, REM SD before the end of the critical period in young rats is known to delay termination of the critical period in the visual cortex (Shaffery et al. 2006). In sleep-deprived young rats, the ratio of serotonin decreases in the hypothalamus (Senthilvelan et al. 2006). This study demonstrates that 48-h of REM sleep deprivation in adolescent rats leads to increased locomotor activity, susceptibility to fear, and induced depression-like behavior. This suggests that poor or altered sleep in adolescence may trigger and maintain many psychiatric and physical disorders or combinations of these conditions, which presumably hinder recovery and may continue into later stages of life. Given the vital importance of sleep, adolescence is a critical period for normal growth and development in which sleep, which has a complex association with many other processes, plays a crucial role. Therefore, timely diagnosis and management of sleep problems appear critical for growth and development, and particularly mental health, in adolescence.
